# A Sequence-Dependent DNA Condensation Induced by Prion Protein

**DOI:** 10.1155/2018/9581021

**Published:** 2018-02-20

**Authors:** Alakesh Bera, Sajal Biring

**Affiliations:** ^1^Infectiologie Animale et Santé Publique, Institut National de la Recherche Agronomique, 37380 Nouzilly, France; ^2^Department of Electronic Engineering and Organic Electronics Research Center, Ming-Chi University of Technology, 84 Gungjuan Rd., Taishan Dist., New Taipei City 24301, Taiwan

## Abstract

Different studies indicated that the prion protein induces hybridization of complementary DNA strands. Cell culture studies showed that the scrapie isoform of prion protein remained bound with the chromosome. In present work, we used an oxazole dye, YOYO, as a reporter to quantitative characterization of the DNA condensation by prion protein. We observe that the prion protein induces greater fluorescence quenching of YOYO intercalated in DNA containing only GC bases compared to the DNA containing four bases whereas the effect of dye bound to DNA containing only AT bases is marginal. DNA-condensing biological polyamines are less effective than prion protein in quenching of DNA-bound YOYO fluorescence. The prion protein induces marginal quenching of fluorescence of the dye bound to oligonucleotides, which are resistant to condensation. The ultrastructural studies with electron microscope also validate the biophysical data. The GC bases of the target DNA are probably responsible for increased condensation in the presence of prion protein. To our knowledge, this is the first report of a human cellular protein inducing a sequence-dependent DNA condensation. The increased condensation of GC-rich DNA by prion protein may suggest a biological function of the prion protein and a role in its pathogenesis.

## 1. Introduction

Cellular prion protein, PrP^C^, is a predominantly *α*-helical soluble glycoprotein which remains attached to the outer membrane of the cells through a glycol-phosphatidyl inositol linkage [1–3]. The protein is noninfectious. The biological role of PrP^C^ remains unclear; however, the protein has been suggested to play a role in a variety of cellular functions including maintenance of cellular copper concentration, signal transduction, RNA binding, and DNA metabolism [4–7]. A beta-sheet rich conformational isoform of PrP^C^, called PrP^Sc^, has been considered to be the major constituent of infectious agent of the fatal neurodegenerative diseases such as Transmissible Spongiform Encephalopathies (TSE) and its hereditary from spongiform encephalopathies (SE) [1–3]. The disease occurs in both human and animals. The disease isoform, PrP^Sc^, exists as oligomers and amyloid polymers and unlike PrP^C^ is resistant to Proteinase K (PK) digestion which is considered as an indicator of formation of PrP^Sc^. Unlike bacterial and viral s where nucleic acid transmits the infection, prion disease has been considered to propagate by the conversion of PrP^C^ by PrP^Sc^ which can occur either by a template or by a nucleation mechanism [2, 8]. The presence of multiple prion strains has also been attributed to the conformational variations of PrP^Sc^. However, the existence of a nucleic acid as a cofactor for infection can explain the strain multiplicity [9–11].

The fibrils formed from* in vivo* isolated hamster PrP 27–30 amyloid or fibrils obtained by converting cellular hamster PrP^C^ have been found to be noninfectious in transgenic mice which overexpress full-length prion protein [12]. However, the amyloid formed from the truncated 90–231 fragment of mouse recombinant prion protein (23–231 amino acid) is found to be infectious in the experimental mice overexpressing this protein fragment and also shows strain characteristics of the prion disease [13, 14]. Inoculation of wild-type hamsters with in vitro generated PK-resistant prion protein formed by protein misfolding has been found to be infectious [15]. By partially disaggregating PK-resistant amyloid isolated from scrapie infected hamster brain, it has been shown that the maximum prion infectivity is associated with prion particles having 17–27 nm diameter (300–600 kDa) whereas the large fibrils show lower prion infectivity [16].

Despite a large number of information favoring infectious agent generated from the protein, the involvement of a slow virus or a nucleic acid in the prion disease has not been ruled out [17–19]. There are several works including our previous data indicating the probable involvement of nucleic acids in the conversion of normal cellular prion protein (PrP^C^) to its pathogenic isoform (PrP^Sc^) [20–22]. Nucleic acids, DNA and RNA in solution and* in vitro*, can catalyze conversion of recombinant and cellular PrP^C^ to PrP^Sc^ (scrapie-like forms) [11, 23–25]. This interaction between recombinant PrP^C^ and nucleic acids produces a mixture of PrP^Sc^ oligomers and linear and spherical amyloids which are similar in morphology to the prion amyloids found in the prion-infected brains [23, 26, 27]. The presence of prion protein in cytoplasm has also been observed [28, 29]. The exact biological significance of prion protein-nucleic acid interaction is not known at present. However, it is possible that structural conversion of PrP^C^ to PrP^Sc^ can be catalyzed by cytoplasmic nucleic acids that can play a role in the prion diseases [30–32]. Our previous study indicated that prion protein can bend the small oligo-nucleotides [21], and this interaction between prion protein- (PrP) nucleic acid was inhibited by biological polyamines [20]. It is also demonstrated that this structural change of prion protein by nucleic acids is pH dependent [22].

The natural polyamines are also known to condense DNA [33], and the sequence-dependent DNA condensation is reported by metal ions [34]. As biological significance, the transcription factor may also interact differentially with GC- and AT-rich chromatin domains [35]. Prion protein is also reported to interact with GC-rich DNA oligomers at a higher order [24, 36]. In addition, our earlier studies also indicated that the herpesvirus capsid protein VP19C is able to condense DNA and forms toroidal DNA structures [37].

In view of the above results on the interaction of prion protein with nucleic acids, we have undertaken a quantitative study of the prion protein interaction with synthetic DNAs by monitoring the fluorescent properties of dye intercalated in nucleic acid bases. We have used human recombinant prion protein, *α*-PrP, and nucleic acids having only GC sequence (gcDNA), only AT sequence (atDNA), and a model DNA (mDNA) containing equimolar ratio of AT and GC base pairs. We also used a 27-mer oligonucleotide which represents a major part of the retroviral stem loop repeat [38]. There are various DNA intercalating dyes which have been used to study the ligand induced structural changes of DNA [39]. Here we have used fluorescent dye YOYO-1 iodide (YOYO), which is a dimer of an asymmetric cyanine dye oxazole yellow YO. YOYO has been used to study the condensation of a plasmid DNA by different DNA-condensing agents and protein NCp7 [40, 41]. Since prion protein is basic, we have compared the prion protein induced structural changes of DNA with the same induced by biological polyamines (such as spermine and spermidine) which are known DNA-condensing agents [42]. Direct ultrastructural studies are also performed to visualize the DNA condensation by pion protein. Images from electron microscope indicated that the gcDNA form a higher order globular condensed structure in the presence of prion protein. Therefore, in this current study we investigated the role of prion protein in condensing DNA with a specificity of GC-contained and its biological role and significance.

## 2. Materials and Methods

### 2.1. Plasmid Construction, Protein (PrP^C^) Expression, and Purification

The recombinant full-length human prion protein (*α*-PrP, 23–232 amino acids, val 129) in reduced form was isolated by following the published procedure as described by Rezaei et al. [43]. The purification procedure utilized the affinity of the octapeptide repeats of the protein in the N-terminal segment for nickel and the use of histidine tag was avoided [43]. In fact, the N-terminal octapeptide region has a high affinity towards different transition metal ions such as nickel and copper [43, 44]. Final protein concentration was measured by the optical density at 280 nm using an extinction coefficient value of 56,795 M^−1^Cm^−1^ [20, 22]. The purified human *α*-PrP migrated in SDS/PAGE as a single species with an estimated molecular mass of 25 KDa. The absence of any high molecular weight band indicated the absence of intermolecular disulfide bond. The identity of the protein was also confirmed by mass spectrometry. The protein strongly reacted with the monoclonal antibody 2D6 (obtained from the French National Antibody Network) specific for human prion protein residues 94–114 [43].

The truncated mouse prion protein spanning 121–231 amino acid (wild-type) was isolated from the plasmid provided by Liemann and Glockshuber using published procedure [45]. The protein in SDS-PAGE experiment showed an estimated molecular weight of 13.5 kDa. An extinction coefficient of 21,000 M^−1^Cm^−1^ at 280 nm was used to calculate the concentration of the protein fragment [20, 22].

### 2.2. DNA Binding Dye and Biophysical Experiments

The dye YOYO-1 was obtained from Molecular Probes. The chemicals and buffers were obtained from Sigma-Aldrich. The synthetic gcDNA, mDNA, and atDNA are consisting 840, 2658, and 3523 base pairs, respectively [22], and purchased from Sigma-Aldrich. The 27-mer oligonucleotide sequence corresponding to a large part of the HIV-1 stem loop region 5′-GACTTGTGGAAAATCTCTAGCATGCAT-3′ and its complimentary strand 3′-CTGAACACCTTTTAGGATCGTACGTA-5′ were also synthesized from Sigma-Aldrich [38]. DNA duplexes were prepared by mixing the complementary oligonucleotides in equimolar amounts (1 : 1) in 10 mM Hepes-KOH (pH 7.5), 100 mM NaCl, 10 mM MgCl_2_ buffer, and hybridized by cooling slowly from 90°C to 20°C over six hours [46]. All experiments were performed in 100 mM NaCl and 20 mM Hepes buffer at pH 7.4. The fluorescence measurements were carried out in a Hitachi 4500 spectrofluorometer equipped with stopped-flow instrument.

### 2.3. Electron Microscopy

The electron microscopy experiments were performed in 0.1 M ammonium acetate, pH 5 at room temperature as described earlier [47]. To monitor the nucleic acid morphology, samples were prepared by the addition of the *α*-PrP (12 *μ*M) to a DNA (5 *μ*M DNA-ph, i.e., ~1 *μ*g/ml DNA concentration) solution in a small tube. After 1 min incubation, one droplet (10 *μ*l) of the specimen was deposited on a carbon coated copper grid followed by blotting of the excess of the specimen on a filter paper. Before drying completely, the grid was washed with 2 droplets of 1.5% w/v uranyl acetate and then washed twice with distilled water [47]. Finally, the grids were observed with a JOEL 1010 electron microscope (fitted with a Gaton digital micrograph) at 80 kV in dark field mode.

### 2.4. Statistical Analysis

The most of the titration (PrP versus different nucleic acids or DNA/PrP with acrylamide) experiments were performed at five individual sets and the averaged data were plotted against the corresponding nucleic acid concentrations. Student's *t*-test (2 tails) was used to compare individual group means. A *p* value of 0.05 was considered as statistically significant. All values in the figures and text were expressed as the mean ± SD.

## 3. Results

### 3.1. The Kinetics of DNA Condensation by *α*-PrP Measured by the Fluorescence Quenching of YOYO Dye Using Stopped-Flow Fluorescence Spectroscopy

**Figure 1:**
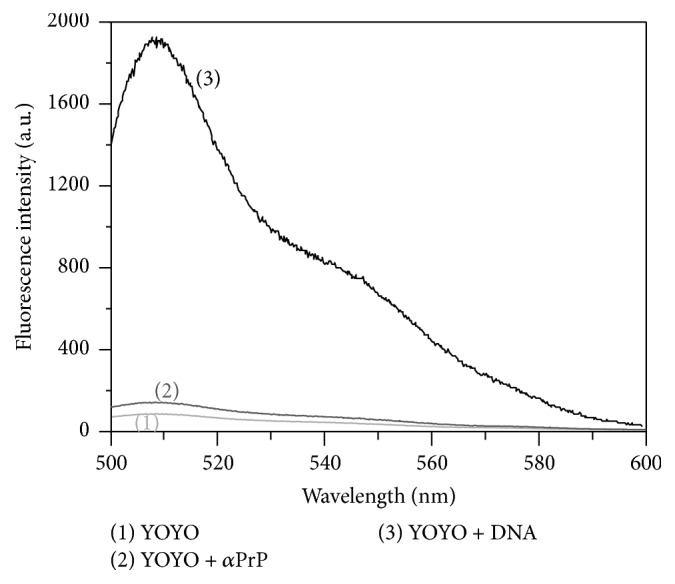
The specificity of oxazole yellow dye YOYO-1 towards DNA. The dye YOYO-1 (at concentration of 10 nM) very specifically binds to or interchelated DNA molecules (mDNA, 1 *μ*M DNA-ph concentration) and increases fluorescence by many folds. YOYO does not bind or increase its fluorescence intensity in the presence of prion protein (*α*-PrP, 2 *μ*M).

**Figure 2:**
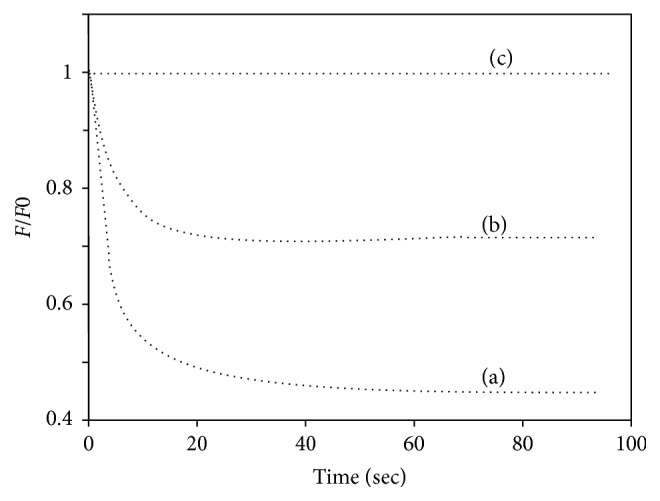
Kinetic studies indicated that the decrease in fluorescence intensity of YOYO intercalated in different DNAs after the addition of 1.2 *μ*M *α*-PrP. Traces (a), (b), and (c) are gcDNA, mDNA, and atDNA, respectively. DNA concentrations 1 *μ*M in phosphate, dye 20 nM (dye to phosphate ratio = 1 : 50). *F*_0_ fluorescence intensity of the dye before the addition of the prion protein and *F* the fluorescence values after the addition of the protein. Temperature 20°C. Excitation 485 nm; emission 505 nm.

The previous study based on electron microscopy generated ultrastructure of prion protein-DNA complexes indicates the formation of globular ordered structure demonstrating the condensation of DNA [47]. Further studies were performed in order to assess and quantify the DNA condensation occurred by recombinant *α*-PrP. To pursue these experiments we used YOYO and which does not fluoresce (or very marginal) in buffer or with recombinant PrP (Figure 1). In the presence of nucleic acids, it shows high fluorescence intensity as a consequence of the intercalation of the dye in the DNA base pairs [40]. In order to establish the time necessary to reach equilibrium between prion protein and nucleic acid interaction by the fluorescence properties of YOYO (20 nM, used this concentration for this experiment) intercalated in nucleic acid (1 *μ*M DNA-phosphate, dye to DNA-phosphate ratio 1 : 50, as described by Krishnamoorthy et al.) [41]. Here, we studied the rate of decrease of fluorescence intensity of YOYO at 505 nm after exciting the solution at 485 nm. The rate of fluorescence quenching of YOYO in the presence of 1.2 *μ*M *α*-PrP is shown in Figure 2. The rate and extent of quenching of the fluorescence in gcDNA (trace (a)) appear greater than those in the presence of mDNA (trace (b)). In both cases, the equilibrium in the fluorescence values of the dye attains a constant value after 20 sec of reaction. The decrease in fluorescence quenching is monophasic. In similar experiment *α*-PrP did not quench the fluorescence of YOYO (or very marginal) intercalated in atDNA (trace (c)).

### 3.2. Sequence-Dependent DNA Condensation Caused by *α*-PrP

**Figure 3:**
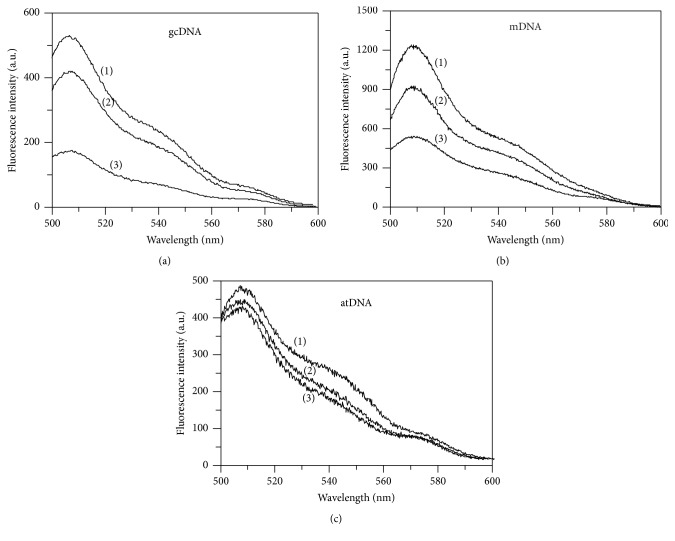
Concentration-dependent effect of *α*-PrP on the emission spectra of YOYO intercalated in gcDNA, mDNA, and atDNA. Traces (1), (2), and (3) in each figures ((a), (b), and (c)) represent the dye fluorescence intercalated in these DNAs, respectively, in 20 mM Hepes buffer, pH 7.4 containing 100 mM NaCl. Traces (2) and (3) are the dye fluorescence spectra after the addition of 0.6 and 1.2 *μ*M *α*-PrP, respectively. DNA and dye concentrations in the experiments were 400 nM phosphate and 8 nM dye. Excitation 485 nm. Temperature 20°C.

The effects of *α*-PrP on the fluorescence spectra of YOYO intercalated in different DNAs are shown in Figures 3(a)–3(c). In these figures, traces labeled (1) represents the fluorescence spectra of the dye in gcDNA, mDNA, and atDNA at a dye to DNA-phosphate ratio of 1 : 50 (8 nM dye and 400 nM DNA-phosphate) in buffer only (absence of protein). It can be seen that, under equal dye to DNA-phosphate ratio, mDNA which contains equimolar amount of AT and GC base pairs induces more than twice the fluorescence intensity of the dye at 505 nm (Figure 3(b), spectrum (1)) compared to the dye spectrum in the presence of both gcDNA (Figure 3(a), spectrum (1)) and atDNA (Figure 3(c), spectrum (1)). A shoulder is also observed in the emission spectra at ~545 nm which is relatively more pronounced in the presence of atDNA. The addition of 0.6 *μ*M and 1.2 *μ*M *α*-PrP to the YOYO intercalated in DNAs reduced the fluorescence intensity of the dye without altering the emission maximum of the dye (spectra (2) and (3) in Figures 3(a)–3(c)). Comparison shows that the recombinant prion protein (1.2 *μ*M) quenches the fluorescence intensity of the dye intercalated in gcDNA, mDNA, and atDNA by approximately 65, 55, and 10%, respectively.

### 3.3. Quantification of the DNA-Structural Change Induced by *α*-PrP

**Figure 4:**
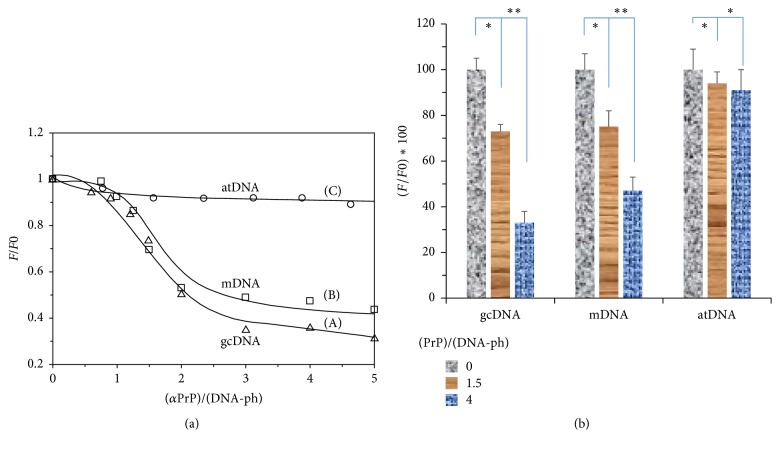
The effect of increasing concentrations of *α*-PrP on the quenching of YOYO fluorescence bound to different DNAs in 20 mM Hepes buffer, pH 7.4 containing 100 mM NaCl. (a) *F*_0_ and *F* are the fluorescence intensities of the dye in the presence of DNA before and after the addition of protein, respectively. DNA and dye concentrations in the experiments were 400 nM phosphate and 8 nM dye. (A), gcDNA, (B), mDNA, and (C), atDNA. Excitation 485 nm; emission 505 nm. (b) A quantitative analysis was also performed to determine the relative quenching of YOYO as a measurement of DNA condensation by PrP. The statistical values were also calculated. *∗∗* represents *p* < 0.01, and *∗* represents *p* < 0.05. Temperature 20°C.

The effects of the increasing concentration of *α*-PrP on the structural properties of different DNAs were monitored from the ratio of the fluorescence intensity values of YOYO bound to DNA in buffer in the absence of protein (*F*_0_) to the fluorescent intensity values (*F*) of the dye bound to DNA in the presence of protein. The results are shown in Figure 4 for gcDNA (A) and mDNA (B) where identical concentrations of both the DNAs (400 nM) were used keeping dye to DNA ratio at 1 : 50. Nonlinear titration curves for the quenching of YOYO fluorescence with increase in *α*-PrP concentration are observed and fluorescence of the dye tends to plateau at protein to DNA-phosphate ratio > 3 for both gcDNA and mDNA (Figures 4(a) and 4(b)). Throughout the titration experiments, the protein induced greater fluorescence quenching of the dye intercalated in gcDNA compared to mDNA. The dye fluorescence was marginally quenched (~7%) when the protein was added to the dye in atDNA and remained virtually unchanged at higher concentrations of proteins where ~70% and 55% fluorescence quenching of the dye occurred in gcDNA and mDNA, respectively (Figure 4).

### 3.4. N-Terminal Domain but Not the C-Terminal Domain of *α*-PrP Is Responsible for the DNA Condensation

**Figure 5:**
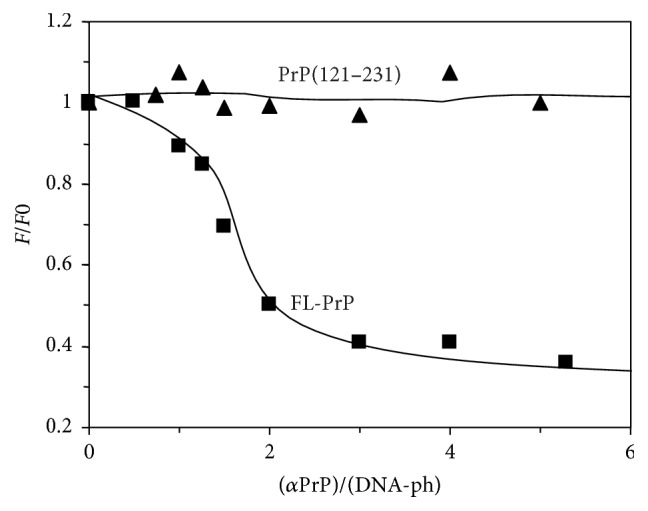
Comparison of the effects of *α*-PrP (trace (a)) and C-terminal PrP 121–231 fragment (trace (b)) on the fluorescence property of YOYO intercalated in mDNA. *F*_0_ and *F* are the fluorescence intensities of the dye in the presence of DNA before and after the addition of protein, respectively, in 20 mM Hepes buffer, pH 7.4 containing 100 mM NaCl. Dye to DNA-phosphate 1 : 50. Excitation 485 nm; emission 505 nm. Temperature 20°C.

The prion protein contains N-terminal unstructured segment (23–120 amino acid residues) which is highly basic and C-terminal globular segment 121–231 amino acids containing three *α*-helices and two short *β*-strands. The globular 121–231 fragments of human and mouse prion proteins have ~95% sequence homology. In order to evaluate the role of this structured part in the conformational change of nucleic acid, we carried out similar experiments as above by adding different concentrations of moPrP 121–231 fragment to the mDNA labeled with YOYO (Figure 5). The addition of the globular C-terminal domain of prion protein (PrP (121–231)) to the DNA is unable to alter the fluorescence property of the dye (YOYO). It is indicated that the globular fragment of the protein does not induce any change in the DNA structure, that is, unable to condense the DNA molecule (Figure 5). For comparison, titration result of *α*-PrP with mDNA (Figure 5) has also been shown. The results of the effect on the YOYO intercalated in gcDNA in the presence of moPrP 121–231 fragment were identical (data not shown).

### 3.5. Condensation Status of Double-Stranded Small Oligonucleotides

**Figure 6:**
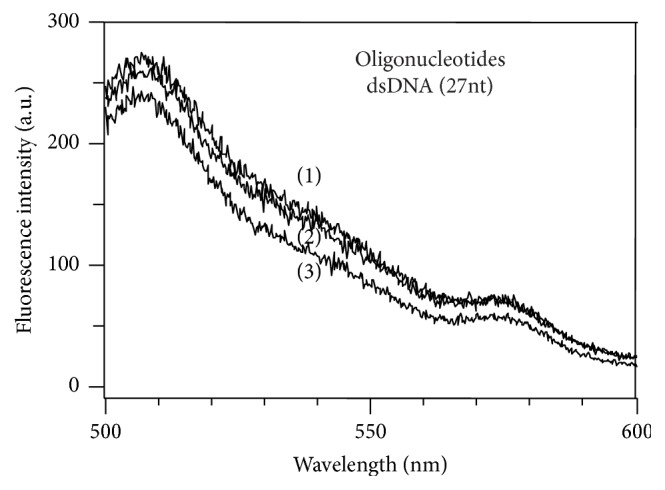
Effect of *α*-PrP concentrations on the emission spectra of YOYO (8 nM) intercalated in 27-mer oligonucleotide (400 nM in phosphate). Spectrum (1), fluorescence spectra of the YOYO bound to the oligonucleotide in Hepes buffer, pH 7.4 containing 100 mM NaCl in the absence of *α*-PrP; traces (2) and (3) are the fluorescence spectra of the dye after the addition of the protein (final concentrations 0.6 and 1.2 *μ*M, resp.) to the oligonucleotides. Dye 8 nM and DNA-phosphate 400 nM (ratio 1 : 50). Excitation 485 nm. Temperature 20°C.

It is apparent from the results that *α*-PrP was unable to induce significant changes of YOYO fluorescence intercalated in atDNA having 3523 base pairs. Since oligonucleotides less than 300 base pairs are not condensed by nucleic acid condensing agents [48], we studied the properties of YOYO fluorescence intercalated in short oligonucleotides. The results in Figure 6 show that the fluorescence of YOYO intercalated in the 27 nucleotide long oligonucleotides decrease by a relatively small amount (~5%).

### 3.6. Spermidine and Spermine as DNA-Condensing Agent

**Figure 7:**
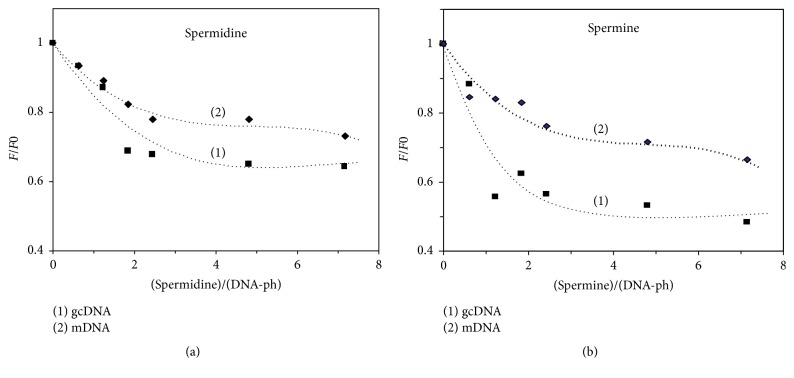
The effect of increasing concentrations of spermine and spermidine on the quenching of YOYO fluorescence bound to gcDNA and mDNA. *F*_0_ and *F* are the fluorescence intensities of the dye in the presence of DNA before and after the addition of amines, respectively. (a) Spermidine. (b) Spermine. (1) and (2) are the results in gcDNA and mDNA in Hepes buffer, pH 7.4 containing 100 mM NaCl. Dye to DNA-phosphate ratio 1 : 50. Fluorescence setup: excitation, 485 nm; emission 505 nm. Temperature 20°C.

Biological polyamines, spermidine, and spermine (carries 3 and 4 positive charges, resp.) are known as condensing agent for DNA structure [42]. It is observed that both amines quench the fluorescence of YOYO intercalated in mDNA and gcDNA (Figure 7) while spermine is more effective in quenching dye fluorescence than spermidine. It has been found that, with spermine or spermidine, the extent of reduction in the dye fluorescence is larger in gcDNA than in mDNA (traces (1) and (2), resp.).

### 3.7. Extent of DNA Condensation Is Measured by the Dissociated YOYO

**Figure 8:**
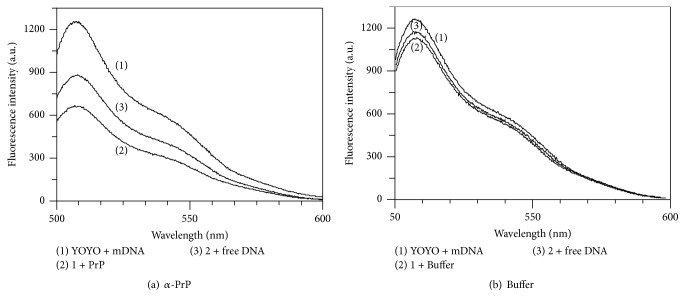
Quantification of the dissociation of YOYO from DNA during its condensation by the prion protein. (a) A 500 *μ*l solution containing YOYO-1 bound to gcDNA (DNA-phosphate 1 *μ*M; dye to DNA 1 : 50) yields the spectrum (1). The addition of 38 *μ*l of stock *α*-PrP (40 *μ*M) to the solution (final protein concentration 3 *μ*M) after 1 hr incubation yields the spectrum (2). Following this, 4 *μ*l gcDNA having 150 *μ*M phosphate concentration was further added to the solution resulting in 1 *μ*M free DNA concentration which yielded the spectrum (3). (b) Control experiment. Spectrum (1) is YOYO bound gcDNA as in (a); (2) and (3), respectively, are the spectra after the addition of 38 *μ*l buffer (instead of protein as in (a)) followed by the addition of gcDNA having 1 *μ*M final DNA concentration.

The possibility of dissociation of YOYO from DNA during its condensation by the prion protein has also been explored. We considered that any dye dissociated from the protein-DNA complex would be present in the bulk solvent which would be nonfluorescent and addition of fresh DNA to the solution would bind to the released dye and increase the dye fluorescence. For this we used the solution containing threefold higher protein compared to gcDNA phosphate (Figure 4(A)) where nearly saturation of the condensation of the nucleic acid by the protein has occurred. In Figure 8(a), the intensity of fluorescence throughout the fluorescence spectrum (1) of YOYO (1 *μ*M DNA-phosphate, 20 nM dye; dye to gcDNA phosphate ratio as 1 : 50) in buffer decreased in the presence of 3 *μ*M *α*-PrP (spectrum (2)). The fluorescence intensity at emission maximum 505 nm was decreased by 50%. The addition of 1 *μ*M DNA (in terms of phosphate concentration) to this solution increased the fluorescence intensity by 30% (spectrum (3)).

An identical experiment was performed with buffer. The addition of the same volume of buffer as off protein experiment resulted in spectrum (2) (Figure 8(b)), indicating the decrease of the fluorescent intensity by ~10% at 505 nm. Next, addition of 1 *μ*M DNA (phosphate) increased the fluorescence intensity of YOYO (spectrum (3)) by only ~2%. From these results, we conclude that the condensation of the gcDNA by *α*-PrP resulted in the release of a fraction of the dye from the condensed gcDNA to the solvent which bound to the newly added DNA, thereby increasing the fluorescence of the solution (from spectrum (2) to spectrum (3) Figure 8(a)). From a calibration curve it is found that ~20% of YOYO is dissociated from the gcDNA on condensation in the presence of 3 *μ*M concentration of *α*-PrP.

### 3.8. Measuring Accessibility of Interchelated YOYO as the Extent of DNA Condensation

**Figure 9:**
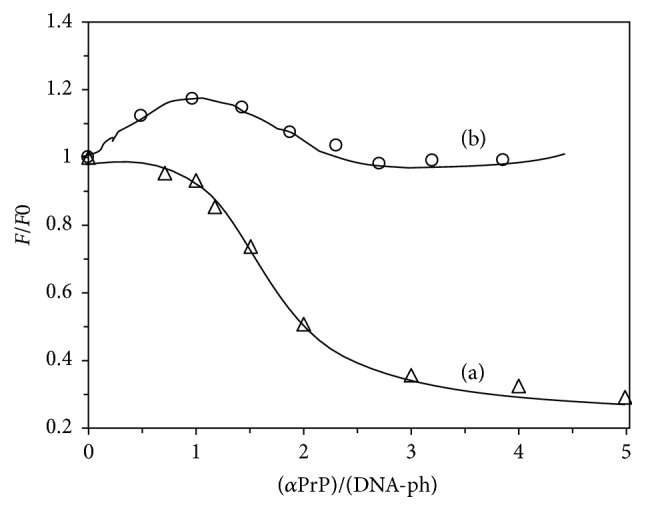
Dependence of fluorescence quenching of gcDNA bound YOYO with varying dye to DNA molar ratio in the presence of increasing *α*-PrP concentrations. (a) Titration with dye : DNA-ph = 1 : 50 and (b) with dye : DNA-ph = 1 : 2000. Therefore dye : DNA-ph ratio = 1 : 2000 used for the acrylamide accessibility experiments.

**Figure 10:**
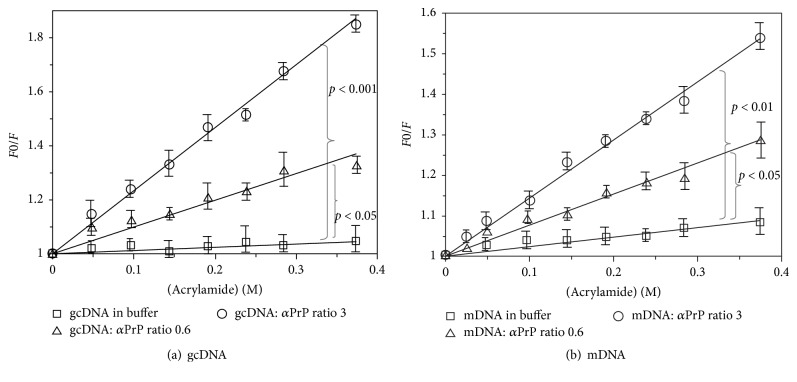
Assess the accessibility of interchelated YOYO as the extent of DNA condensation. Stern-Volmer plots of acrylamide quenching of YOYO bound to gcDNA (a) and mDNA (b) in the presence of different concentrations of *α*-PrP in Hepes buffer, pH 7.4 containing 100 mM NaCl. Concentrations of the DNAs in phosphates were 10 *μ*M and the dye concentrations were 5 nM in both the experiments (dye to DNA-phosphate 1 : 2000). *F*_0_ and *F* are the fluorescence intensities of the dye in buffer and in the presence of increasing concentrations of acrylamide. □, △, and ○ in the figures are the fluorescence quenching results in buffer and in the presence of PrP : DNA ratios of 0.6 and 3, respectively. Five individual titration experiments were performed and the average was plotted against acrylamide concentration. The statistical data were calculated with Student's *t*-test.

The change in the physical states of the DNA bases, namely, the possibility of their exposures to the surrounding solvent in the presence of *α*-PrP, was investigated by monitoring the accessibility of YOYO to a neutral fluorescence quencher acrylamide in the dye-DNA complex with and without the presence of the protein [40, 41]. This procedure needs the monitoring of the property of the individual dye molecule instead of condensation induced interaction between DNA-bound YOYO-1 molecules resulting in the quenching of the fluorescence of the dye as observed with dye to DNA-phosphate (1 : 50) above. We found that, by using low dye concentration (dye to DNA-phosphate ratio as 1 : 2000), addition of prion protein did not quench the dye fluorescence which would arise from dye-dye interaction (Figure 9, trace (b)). The results are shown in Figure 8 where increasing protein concentrations did not quench the YOYO-1 fluorescence intercalated in gcDNA under this experimental condition (trace (b)). Metal ion-induced plasmid DNA condensation having identical dye to DNA-phosphate ratio also did not quench the DNA-bound YOYO fluorescence [40]. Besides, similar kind of acrylamide quenching experiments performed to quantify the macromolecular interaction including prion protein (PrP) and nucleic acids [36]. Based on these, we have used YOYO to DNA-phosphate ratio of 1 : 2000 for acrylamide fluorescence quenching experiments described later (Figure 10). For comparison, we have also shown results when higher concentration (1 : 50 dye to DNA-ph ratio) of the dye was used (Figure 9, trace (a); results taken from Figure 4, trace (A)).

The exposure of YOYO and hence the nucleic acid bases on condensation in the presence of *α*-PrP were determined from the ratio of the values of *F*_0_ and *F*, where *F*_0_ represents the fluorescence intensity of the dye intercalated either in DNA in buffer or in the presence of protein and *F* is the fluorescence value of the dye either in DNA or in the protein-DNA complex in the presence of acrylamide (Figures 10(a) and 10(b)). The data analysis was done by using the Stern-Volmer equation and the slope (*K*sv) of *F*_0_/*F* plot against acrylamide concentrations [*Q*] is linear for the dye intercalated in gcDNA with a slope of 0.11 indicating that the dye is poorly accessible to acrylamide (Figure 10(a), squares). However, in the presence of *α*-PrP at molar ratios of protein to gcDNA phosphates of 0.6 and 3, acrylamide quenched the fluorescence of the dye considerably as evidenced from the values of slopes, namely, 0.99 and 2.33, respectively (Figure 10(a), triangles and circles, resp.). The results indicate ~9- and 23-fold increase in the dye accessibility when above concentrations of protein were added to gcDNA compared to DNA alone (Table 1). The corresponding values of the quenching of the fluorescence of YOYO intercalated in mDNA in buffer and in the presence of above *α*-PrP concentrations are 0.23, 0.76, and 1.43 resp. (Figure 10(b), symbols have the same significance as in Figure 9a). The results indicate approximately 3- and 6.5-fold increase in the accessibility of the mDNA intercalated YOYO in the presence of *α*-PrP at mole ratio of protein to mDNA phosphates 0.6 and 3, respectively. Since the dye is intercalated in the nucleic acid base pairs, the above results suggest that nucleic acid bases in the gcDNA become highly exposed to the solvent compared to mDNA on condensation by the prion protein. In Table 1, *K*sv values obtained from the above plots have been shown. For comparison, similar values for spermine induced condensation of gcDNA have been presented which show that spermine is less effective than the prion protein in exposing the intercalated YOYO in the DNA.

### 3.9. DNA-*α*PrP Ultrastructure Analysis

**Figure 11:**
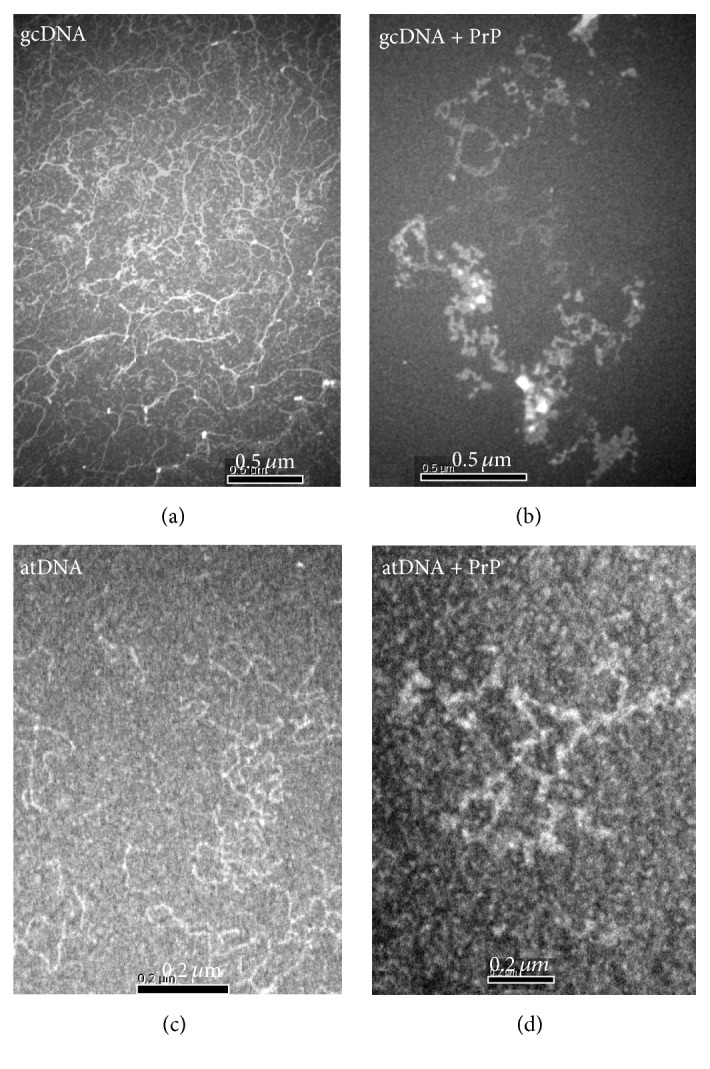
The ultrastructure of DNA-PrP interaction was analysed by scanning electron microscopy (SEM) technology. The electron microscopy generated morphology of gcDNA (5 *μ*M DNA-phosphate) alone and after addition of *α*-PrP (12 *μ*M; 1 minute of incubation) to it ((a) and (b)). The scale bar represents 500 nm. Morphology of atDNA (5 *μ*M DNA-phosphate) alone and morphology of with *α*-PrP (12 *μ*M) after 1 minute of incubation ((c) and (d)). The scale bar represents 200 nm. A clear condensed gcDNA structure was observed in the presence of *α*-PrP (b).

To visualize and measure the extent of DNA condensation, we also pursue the scanning electron microscopic (SEM) analysis of different DNA molecules with prion protein. The images from SEM system indicated that the DNA molecules with GC nucleotides form higher order globular structures in the presence of *α*-PrP, whereas atDNA they form lower order simple condensed DNA structures (Figure 11).

## 4. Discussion

The dye YOYO-1 when intercalates in DNA shows a large increase in its fluorescence intensity [40, 41]. The differences in the emission intensities of the dye in different DNAs (Figure 3) most probably reflect the differences in the microenvironments in different DNAs where the dye binds [49]. Various DNA-condensing agents have been found to quench the fluorescence intensity of the intercalated YOYO in DNA [50]. From similar observations it has been concluded that NCp7 protein of HIV-1 virus also condenses DNA [41]. The decrease in YOYO fluorescence intensity arises from the condensation of the DNA resulting from the binding of the condensing agents to the DNA. Mechanistically the quenching of the YOYO fluorescence intensity has been explained to arise from both the formation of the dye dimer and fluorescence resonance energy transfer between the dimers and monomers of the YOYO in the condensed state of DNA [40, 41]. From these considerations we believe that *α*-PrP induces condensation of the nucleic acid containing only GC and GC and AT base pairs. However a small percentage of the decrease in the fluorescence intensity of the dye also results from the dissociation of the dye intercalated in nucleic acid condensed structure in prion protein-nucleic acid complex (Figure 8). A similar kind of dye dissociation also occurred during condensation of a plasmid DNA by spermine [34, 51].

Spermine and spermidine are biological amines which have been shown to condense DNA by various physicochemical methods [34, 51]. It has been found that spermine containing four positive charges is more effective in condensing DNA than spermidine which carries three positive charges and this is reflected in the present study. However, it is important to note that after a long period of incubation these amines induce rod and toroid structured polymers of DNA whereas prion protein induces ordered globular aggregates of DNA molecules [42, 47]. The present results also show that *α*-PrP which carries overall seven positive charges is more effective in condensing the DNAs than the biological amines suggesting that electrostatic interaction probably plays a dominant role in DNA condensation.

The prion protein is found to condense the gcDNA faster than mDNA as evidenced from the initial decrease of the YOYO fluorescence after the addition of the protein. In the kinetic experiments, the time for fifty percent reductions in dye fluorescence by prion protein is ~3 and 5 secs for gcDNA and mRNA, respectively (Figure 2). The extents of the reduction of YOYO fluorescence by the protein induced gcDNA and mDNA condensation were ~50% and <30%, respectively. Therefore the extent of prion protein induced condensation is greater for gcDNA than mDNA. The results also show that DNA containing only AT sequence unable to condense in the presence of prion protein even after a long period of equilibration (Figure 2). The spectra of the dye after the attainment of equilibrium condensation also lead to similar conclusion (Figures 3(a)–3(c)). The condensation reaction follows a transition between protein to nucleotide mole ratio 1 to 3 for both the gc and mDNA (Figure 4). Previous ultrastructure studies indicated that the full-length recombinant *α*-prion protein induces ordered aggregation of both GC and mDNA molecules to condensed globular structure [47]. Our current study also indicates that the gcDNA forms higher order condensed globular structure in the presence of prion protein.

A comparison of the results presented in Figure 5 shows that the globular 121–231 fragment of the prion protein, which contains the ordered structures (three *α*-helices and two beta-strands), does not condense nucleic acid. Therefore, the N-terminal unstructured highly basic 23–120 segment containing seven lysine, three arginine, and six histidine residues is responsible for the condensation. Although full-length prion protein and its 121–231 fragment bind nucleic acids with comparable affinities (unpublished results), the inability of the protein fragment to condense nucleic acids suggests that condensation of the DNA is independent of the binding energy between the protein and nucleic acid. Similar conclusion has been arrived from the DNA-condensing property of various NCp7 peptides [41]. The previous result showed that unstructured N-terminal highly basic segment and not the structured region (C-terminal) of prion protein is involved in the functional properties of the protein [52]. It is also important to mention that a recent study demonstrated the unstructured N-terminal domain of prion protein containing a polycationic cluster (KKRPKPG) similar to the GPRGKPG motif of the Gpr126 agonist type IV collagen2 [53]. Besides, study also indicated that a KKRPKPG-containing PrP^C^ derived peptide is sufficient to induce a Gpr126-dependent cAMP response in cells and mice and improved myelination in hypomorphic gpr126 mutant zebrafish [53].

It is known that oligonucleotides having less than 300 base pairs are not amenable to condensation by any DNA-condensing agent [42, 48]. This can explain the relatively small decrease (10%, Figure 5) in quenching of fluorescence of YOYO intercalated in the 27-mer oligonucleotides by the prion protein compared to quenching of YOYO fluorescence of ~65% and 55% in gcDNA and mDNA, respectively, at identical protein to nucleotide mole ratio.

The extent of base exposures by acrylamide quenching of the fluorescence of intercalated YOYO in nucleic acids shows that among the nucleic acids studied here, bases of atDNA are exposed to a maximum extent in buffer whereas those in gcDNA are least exposed in the absence of prion protein. The bases in mDNA are exposed to an intermediate extent. The poor solubility of guanine base in water compared to other bases can explain the least exposure of the GC base pairs in surrounding solvent. Despite this unfavorable energetics, *α*-PrP induced condensation exposed the GC bases of gcDNA more than those of mDNA containing both AT and GC bases. The extent of base exposure of atDNA by the protein was minimum among the three nucleic acids studied and the extent of base exposure did not change with the increase in protein concentration (above a mole ratio > 3, Table 1). These results show that the structure of gcDNA is destabilized to a greater extent by the prion protein compared to the other two DNAs.

Therefore, it is important to ask the question, how compact is the DNA condensed by PrP? In a similar experiment, the accessibility of the YOYO in the condensed plasmid DNA by NCp7 of HIV-1 towards acrylamide quenching experiment showed *K*_SV_ = 0.15 M^–1^; and branched polyamine (PEI) showed *K*_SV_ = 0.6 M^−1^ [41]. In this current study, we observed that the DNA condensation induced by *α*-PrP (*K*sv = 2.32 M^−1^ for gcDNA) is much higher than the NCp7 of HIV-1. The current observation indicated that the extent of reduction in the fluorescence intensity of YOYO-1 (Figures 3 and 4) on condensation by *α*-PrP is around 65% (gcDNA) and 55% (mDNA), which is more than 50% and 30% reduction, respectively, observed with spermine (Figure 7). Therefore, the condensation or compactness of DNA occurred by prion protein is much higher than the biological polyamines or HSV-1 protein of NCp7.

It has been found that Ni^2+^ ion condenses gcDNA significantly and has only very mild condensing effect on atDNA [34]. Therefore our results of prion protein condensing most efficiently gcDNA and without any significant effect on atDNA represent properties of the nucleic acids which are composition and sequence specific. It has also been found that, in the presence of cations, condensation of plasmid B-DNA is enhanced by the insertion of d(CG)*n* sequence [54]. Secondary structures of the DNAs studied by circular dichroism show that the Ni^2+^ ion converts gcDNA to Z-form from its B conformation but shows marginal effect on atDNA structure [34]. Ni^2+^ ion condenses gcDNA extensively whereas atDNA is condensed to a very small extent which shows that Z-form of DNA is more prone to condensation compared to other structural forms of DNA. Biological polyamines converts B-form of DNA to its Z-structured form determined by different experimental approaches [55–57]. Spermine and spermidine are found to condense nucleic acid by the method used in the present study and induce exposure of nucleic acid bases from the interior of the molecule (Table 1). The major groove of Z-DNA with the sequence (GC)_8_ and B-DNA features a narrow minor groove and a broad major groove [58]. Our result indicated that during this sequence specific DNA condensation process prion protein may interact with the major groove of the B-DNA. This observation is also supported by the previous study which includes the nonspecific interactions between prion protein-nucleic acid [36].

What are the origin and importance of this sequence-dependent DNA condensation by prion protein? In order to get the answer, we need to consider the interaction between calf thymus DNA and a tandem repeat of the SPKK peptide motif of histone H1. The interaction between basic octapeptide SPKKSPKK of Histone H1 and the alternating copolymer poly(dA-dT)-poly(dA-dT) results in Ψ-type condensation [59]. The N-terminal domain of prion protein contains a polycationic cluster (KKRPKPG) and study indicated that a KKRPKPG-containing PrP^C^ derived peptide is sufficient to induce a Gpr126-dependent cAMP response in cells [53]. It is possible that the high content of glycine molecules in the N-terminal octapeptide repeats (PHGGGWGQ) of prion protein differentially interacts with the DNA backbone. Glycine residues are known as biological osmolyte and N-terminal of prion protein able to interact as a chaperon with macromolecules such as protein and DNA [52, 60–62]. It is also important to mention that the PrP is able to interact with nuclear DNA at both normal physiological or pathological conditions [63, 64]. It is possible that, in the prion related neurodegeneration, the prion protein and nucleic acid interaction may lead to amyloid aggregation. In the case of cancer, p53 aggregation appears to sustain the proliferative nature of tumors [64], whereas aggregation in neurodegenerative diseases leads to cell death, although the proteins appear to share the same mechanisms for prion-like conversion. Interestingly, aggregation occurs by formation of hetero-oligomers containing mutated-p53 with altered DNA binding activity [64].

In terms of interaction between histone and the different domains of chromatin, the GC-rich and AT-rich chromatin domains display distinct chromatin conformations and are marked by distinct patterns of histone modifications. The previous study identified the histone deacetylase Rpd3p as an attenuator of these base composition-dependent differences in chromatin status [35]. The result also indicated that the GC-rich chromatin domains tend to occur in a more active conformation and that Rpd3p activity represses this propensity throughout the genome [35]. Recent study also indicated that, in DNA-DNA interaction, the AT-rich DNA duplexes associate more strongly than GC-rich duplexes [65]. Besides, methylation of cytosines makes attraction between GC-rich DNA as strong as that between AT-rich DNA. Therefore, it is suggesting that the AT-rich methylated DNA-DNA interactions may play a critical role in the chromosome organization and gene regulation [65]. Prion protein has been demonstrated to be present in the cytoplasm [29, 30]. Internalization of PrP^C^ in neuronal cells in the presence of copper ions shows that the protein is present in the perinuclear compartment as well [66]. It has also been demonstrated that full-length prion protein without the GPI anchor accumulates in the nucleus of the hippocampus and neuroblastoma cell lines which would suggest interaction of the prion protein with the chromatin [67]. The binding of prion protein to the chromatin may suggest a biological function of the protein. Telomeres at the end of the chromosomes are rich in GC sequence which becomes shorter with cell divisions. Shorter telomeres are known to favor end-to-end fusion of chromosomes [68, 69]. Prion protein induced stable condensed structure observed with GC-rich DNA. It is possible that the current PrP mediated condense DNA morphology resembles with telomeres structure. The condensed telomeres can suppress end-to-end chromosome fusion by inhibiting DNA base pairing between chromosome ends. Therefore, all these data indicate that prion protein may be involved in chromosome organization and gene regulation.

Proteinase K-resistant PrP^Sc^ has been found to accumulate in the nuclei of prion-infected cells and is associated with chromatin independently of proteasome inhibition [70, 71]. The protein concentrates in euchromatin regions that correspond to a highly transcribed part of the chromosome. In genome, the major transcription region contains high concentrations of GC sequence [72]. It has also been observed that chromosomal regions with higher recombination rate have higher GC content. It is a matter of speculation whether the specific interaction of the prion protein with GC-rich segment in the genome would influence gene expression which can ultimately result in prion pathogenesis.

It is assumed that the prion protein-nucleic acids complex regulates different cellular functions which correlate with the biophysical properties of the complex. Previous studies indicated that there may be sequence specific interaction between GC-rich oligonucleotide and prion protein [24, 36]. It is important to mention that the sequence specificity is not obvious for prion protein-nucleic acid interactions. Rather, it is assumed that the particular nucleic acid patterns (DNA/RNA), possibly related to GC composition, oligonucleotide length, and structure, define the prion protein recognition and pattern of interaction. However, current study shades light on this prion protein induced sequence-dependent DNA condensation and interprets the probable origin, consequence, and biological significance. Understanding the structural and cellular effects observed in PrP–DNA complexes may reveal the mysterious pathology of the prion protein related malignancies. The results presented in this work are extremely relevant to building models for understanding the role of prion protein towards compactness of DNA in chromosomes and regulating gene expression.

## Figures and Tables

**Table 1:** Stern-Volmer constant *K*sv for the accessibility of YOYO bound to different DNAs in the presence of *α*-PrP or spermine in Hepes buffer, pH 7.4 containing 100 mM NaCl.

DNA molecules	Base pair	*K*sv mol^−1^ for [PrP]/[DNA-ph] or [spermine]/[DNA-ph]		
		0	0.6	3
gcDNA	840	0.11 ± 0.05	0.99 ± 0.35	2.32 ± 0.58
mDNA	2658	0.23 ± 0.04	0.76 ± 0.24	1.43 ± 0.13
atDNA	3523	0.44 ± 0.09	0.81 ± 0.27	0.81 ± 0.21
Spermine	With gcDNA	0.11 ± 0.05	0.64 ± 0.21	0.84 ± 0.19
